# 1003. Cytokine Levels in Sepsis and TNFα Association with Mortality but not Sepsis Severity or Infection Source: a Systematic Review and Meta-analysis

**DOI:** 10.1093/ofid/ofab466.1197

**Published:** 2021-12-04

**Authors:** Amal Gharamti, Omar Samara, Anthony Monzon, Lilian Vargas Barahona, Sias Scherger, Kristen DeSanto, Daniel B Chastain, Stefan Sillau, Carlos Franco-Paredes, Andrés F Henao Martínez, Leland Shapiro

**Affiliations:** 1 Department of Internal Medicine/American University of Beirut, Beirut, Beyrouth, Lebanon; 2 University of Colorado Denver/School of Medicine, Aurora, Colorado; 3 Department of Medicine, Division of Infectious Diseases, University of Colorado Denver, Aurora, Colorado; 4 Health Sciences Library, University of Colorado Denver, Aurora, Colorado; 5 University of Georgia College of Pharmacy, Albany, GA; 6 University of Colorado Denver, School of Medicine, Aurora, Colorado; 7 University of Colorado Anschutz Medical Campus, Aurora, Colorado

## Abstract

**Background:**

Sepsis is a global health problem associated with significant morbidity and mortality and is attributed to a “cytokine storm.”. However, anti-cytokine therapies have failed to lower sepsis mortality in clinical trials. Linking cytokine excess to sepsis pathogenesis requires quantification of cytokine levels in sepsis. This systematic review and meta-analysis characterizes levels of key cytokines in the circulation of sepsis patients and relates TNFα levels to mortality and patient characteristics.

**Methods:**

Medline, Embase, Cochrane Library, and Web of Science Core Collection databases were searched from 1946 to May 2020 for studies in English disclosing cytokine levels in sepsis. Keywords included sepsis, septic shock, purpura fulminans, and tumor necrosis factor (TNF)α. We related cytokine amounts to 28-day mortality. Data analyses were performed using a random-effects model to estimate pooled odds ratios (OR) and 95% confidence intervals (CI). This systematic review is registered in PROSPERO under number CRD42020179800.

**Results:**

A total of 3656 records were identified. After exclusions, 103 studies were included. Among these studies, 72 disclosed TNFα levels, 25 showed interleukin (IL)-1β levels, and 6 presented interferon (IFN)γ levels. The pooled estimate mean TNFα concentration in sepsis patients was 58.4 pg/ml (95% CI, 39.8-85.8 pg.ml; I^2^ = 99.4%). Pooled estimate means for IL-1α and IFNγ in sepsis patients were 21.8 pg/ml (95% CI, 12.6-37.8 pg.ml; I^2^ =99.8%) and 63.3 pg/ml (95% CI, 19.4-206.6 pg/ml; I^2^ = 99.7%), respectively. Elevated TNFα concentrations were associated with increased 28-day mortality (P=0.001). In a subgroup analysis, TNFα levels did not relate to sepsis source, sepsis severity, or sequential organ failure assessment (SOFA) score (figure 1). In a metaregression, TNFα associated with age, percentage of females and mortality at 28 days.

Figure 1: A: TNFa levels according to sepsis source. B: TNFa levels according to measurement technique. C: TNFa levels according to presence or absence of cardiovascular disease. D: TNFa levels according to presence or absence of malignancy. E: TNFa levels according to sepsis severity. F: TNFa levels in fungal compared to other causes of sepsis (Yes=fungal sepsis; No= Other types of sepsis). G: TNFa levels according to SOFA score. H: TNFa levels and mortality at 28 days.

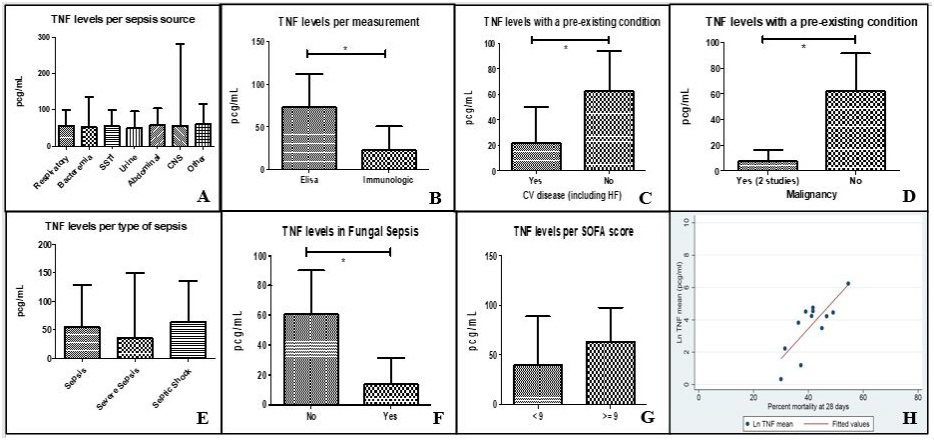

**Conclusion:**

We presented levels of TNFα, IL-1β, and IFNγ in human sepsis and showed that TNFα elevations are associated with sepsis mortality. TNFα concentrations did not correlate with sepsis severity. We believe the concept that elevated cytokines cause sepsis should be revisited in the context of these data.

**Disclosures:**

**All Authors**: No reported disclosures

